# Constructing respectability from disfavoured social positions: exploring young femininities and health as shaped by marginalisation and social context. A qualitative study in Northern Sweden

**DOI:** 10.1080/16549716.2018.1519960

**Published:** 2018-10-01

**Authors:** Maria Wiklund, Christina Ahlgren, Anne Hammarström

**Affiliations:** a Department of Community Medicine and Rehabilitation, Physiotherapy, Umeå University, Umeå, Sweden; b Department of Public Health and Caring Sciences, Public Health, Uppsala University, Uppsala, Sweden

**Keywords:** Youth, femininity, gender, femmephobia, respectability, agency within structures, youth unemployment, public health, qualitative interviews, Sweden

## Abstract

**Background**: Gender, class and living conditions shape health and illness. However, few studies have investigated constructs of femininity in relation to health and living conditions among young women who are unemployed and marginalised at an early age.

**Objective**: The aim of this research was to elucidate constructs of femininities in relation to structuring living conditions and expressions of health in Northern Swedish women. The time period of interest was the transition from unemployed teenagers to young adults in a social context of high unemployment and societal change across the critical ‘school-to-work-transition’ period of the life course.

**Methods**: Qualitative content analysis was used to analyse data from repeated interviews with unemployed young women, aged 16–33 years, during the 1980s and 1990s. These longitudinal interviews were part of a cohort study in a ‘remote’ municipality in Northern Sweden that began in 1981. All girls who were not in education, employment, or training were selected for interview. An inductive analysis phase was followed by a theoretically informed phase. The contextual frame is the Nordic welfare-state model and the ‘caring state’ with its particular focus on basic and secondary education, and women’s participation in the labour market. This focus paralleled high rates of youth unemployment in northern Sweden during the study period.

**Results**: The results are presented as the theme of ‘constructing respectability from disfavoured social positions’. Within this theme, and framed by dominant norms of patriarchal femininity, we explored the constructs of *normative and altruistic, norm-breaking*, and *troubled* femininity.

**Conclusions**: Gender-sensitive interventions are needed to strengthen young women’s further education and positions in the labour market and to preventing exposure to violence. More research on health experiences related to the multitude of constructs of femininities in various social contexts and across the life course is needed to help design and implement such interventions.

## Background

Gender theoretical research is needed to understand marginalised and unemployed young women’s poorer health and vulnerable positions in society [1]. In line with Beauvoir [2], and linking these positions to health, Springer et al. [3] emphasise that ‘gender is made, not born’ and that ‘gender is inescapably embedded in and constitutive of social structure’ (pp. 1662–1663). According to Annandale [4], and consistent with our own view, women’s health should be understood in relation to the social and gendered context. Seeking to understand how constructs of gender and femininity shift over the life course is important [5]. A cornerstone within this framework of gender relations is that femininities and masculinities are constructed in the context of a gender order where male domination is created and maintained [6,7]. Graham [5] argues that young women’s options for negotiating various femininities become constrained during the transition from school into adulthood, and this has health consequences such as risky sexual behaviours.

In analysing constructions of femininities and health, addressing variations *within* the group of women is of central importance because femininity positions in various social contexts are plural, dynamic, diversified, and informed by both individual agency and collective patterns [8–13]. As background, Jewkes and Morell [8] state that health literature, epidemiology, and qualitative research have tended ‘to treat women as a homogeneous group and as victims of (all) men’ and deployed the categories woman or female ‘in a blunt and uncritical manner’ instead of exploring their agential capacities (p. 1730). Moreover, addressing issues of power are crucial [14], including the more general and systemic devaluation of femininity across genders and sexualities [7,15]. Intersectional approaches have the potential to capture interlinked ‘axes of oppression’, including dominant norms of ‘whiteness’ and ‘heteronormativity’ [7,16–18].

While gender perspectives have become more common in health research [19], public health research rarely focuses on how processes of constructing femininities come into play. Few studies have investigated femininity in relation to health and living conditions among young marginalised women, or the important transition from adolescence into adulthood.

The aim of this research was to elucidate constructs of femininities in relation to structuring living conditions and expressions of health in Northern Swedish women. The time period of interest was the transition from unemployed teenagers to young adults in a social context of high unemployment and societal change across the critical ‘school-to-work-transition’ life course period.

Repeated interviews were drawn from a cohort and conducted from the early 1980s until the late 1990s. This period represented an important transition phase between school and employment for these participants. This period also represented a socio-political breakpoint in the Swedish welfare state with a specific focus on women’s participation in the labour market and emancipation through work [20].

### Through the lens of femininity and respectability: young working-class women in context

Our study is framed by the assumption that individual agency, gender constructs, and experiences of health and illness are both discursively and materially shaped in relation to macro-level conditions and policies at a certain time and place. This refers to a wider ‘agency within structure’ framework in which health and illness are integral aspects [21–23]. Within this framework, we understand gender as ‘embodied structure’ in which gender is seen as ‘the active social process that brings reproductive bodies into history, generating health consequences not as a side-effect but in the making of gender itself’ [24] (p. 1675). Thus, a relational and bio-social constructionist view is applied to gender, as well as health and illness [3]. Like Cosgrove [25], we view women’s emotional distress as both lived experience and social constructs. According to Weitz [26], ‘social constructions’ refers to the process through which ideas become socially accepted, and this political process and these constructs reflect competing groups’ different interests and access to power (p. 1). Throughout history, socially accepted ideas about women’s bodies have affected ‘the strictures within which women live’ (p. 10). Weitz [26] argues that by looking at women’s embodied experiences and how these are socially constructed, we can understand more about ‘women’s position in society, and the possibilities for resistance against that position’ (p. 10).

Femininity is described by Skeggs [27] as the process ‘through which girls and women are gendered and become specific forms of women’ (p. 98). Processes of ‘doing’ femininity are seen as context-bound, and may change with historical and political context [28]. As Graham [5] notes, constructs of femininity may ‘shift over the life course as young people are exposed to different social and cultural contexts’ (p. 266). And, there is more than one way to construct, navigate and negotiate femininity, which can be dependent on place, time and micro-politics of life [5,8–10,13,15,17,29–35]. Performances and practices of femininity are thus understood as ‘social constructions’ intrinsically linked to health and illness [10,36].

In this study, *normative femininity* is defined as the culturally dominant femininity that has been constructed through history as the ‘normal’ and therefore ‘naturalised’ way to adequately perform western and ‘white’ femininity. Ringrose and Renold [34], as well as other scholars, describe the idealised cultural expectations for girls (‘the normative subject position girl’) to perform ‘niceness’. This means being good, caring, nurturing, sexually innocent, and respectable, as well as being ‘supportive’, ‘non-competitive’ and ‘there for you’ (p. 1730). Connell [37] terms this ‘emphasised femininity’. Emphasised femininity may be understood as tied to traditional gender orders of women’s responsibility and caring within domestic fields [10] (p. 1571), and characterised by ‘compliance with women’s subordination and accommodation of the interests and desires of men’ [8] (p. 1730). Normative femininity is commonly described as performed within the context of what Rich [38] terms ‘compulsory heterosexuality’, and Butler [39] terms ‘the heteronormative matrix’.

However, the concept of femininity is not neutral and is thus problematised. Critical femininity studies, intersectional feminist approaches, and ‘queer fem(me)inist’ scholars question the understandings and theories of femininity that are limited to white, cisgender, heterosexual, female (able) bodies [7,12,15,17,40,41]. As pointed out by Hoskin [7] in concepts on femme theory and intersectionality, and Dahl and Sundén [41], ‘hegemonic’, ‘normative’ or ‘patriarchal’ femininity is not only tied to dominant gender norms, but also to class (upper-middle), race/ethnicity (whiteness), ability (able-bodied) and sexuality (heterosexuality). According to Deliovsky [17], the white patriarchal discourse and *normative white femininity* represent white women as the ‘benchmark woman’. This is illustrated in the context of beauty-ideals. She describes this as ‘a hegemonic ideology and social location that define dominant and subordinated femininities’ (p. 49). Deliovsky [17] not only talks of compulsory heterosexuality [38], but also about compulsory *white* heterosexuality, as required in the context of heteronormativity for white women in white patriarchy.

According to Skeggs [27], *respectability* is ‘one of the most ubiquitous signifiers of class’, something ‘to desire, to prove and to achieve’ (p. 1), and thus is not a given. ‘Becoming respectable’ is conceptualized by Skeggs [27] and Sohl as a key mechanism in women’s formation of classed and gendered social positions. Hatherley [42] states that femininity is historically a concept formed by structures of class and racial difference; to be ‘feminine’ is to fit into an idealised, higher-class position. Working-class women without the financial resources to successfully perform femininity or to give the correct presentation of femaleness, are regularly ‘cast down into the realms of the grotesque’ (pp. 3–4). This ‘fall from grace’ has repercussions on the representation and lived experiences of women who are then defined negatively (pp. 3–4). Hatherley argues that working-class women are subject to intersections of sexist and classist oppression by being defined against the ‘acceptable’ forms of feminine presentation (pp. 3–4). According to Dahl [41], with reference to Skeggs [27], the default mode of ‘respectable’ and idealised heteronormative femininity is *failure*. And the risk of failure is tied to class (not bourgeois) and race (not white). Further, Blair and Hoskin [15] argue that ‘culturally sanctioned’ and ‘essentialised’ femininity consists of the Victorian model of ‘proper womanhood’ that ‘necessitates [a] white, heterosexually available, cis woman’, and espouses a biological determinist view of gender (p. 232).

Central in theorising about the multitude of femininities is the issue of ‘policing’ and ‘disciplining’ of femininity. This includes self-regulation and self-surveillance in order to conform to normative femininity and respectability, and is seen in the context of appearance ideals and the use of body technologies [7,10,17,43,44]. Bartky [44] utilises a Foucauldian perspective on ‘docile bodies’ to problematise the often internalised disciplinary body-practices used to conform to ‘properly’ embodied femininity and heterosexual appearance ideals. Bordo [43] emphasises that the body is crucial for the reproduction of femininity, as a ‘medium of culture’, and is central to understanding classed and gender-related, historically localised disorders like hysteria and anorexia nervosa.

In relation to femme theory, the concept of femmephobia is used to theorise ‘naturalised’ policing, stigmatisation and subordination of femininity in relation to the privilege and dominance of masculinity across genders and sexualities [7,15]. Hoskin [7] describes ‘femme’ as an ‘active subject’ and a ‘failed femininity’ who has failed or refused to ‘approximate the patriarchal feminine norm of white, cisgender, able-bodied virtuosity’ (p. 100), while ‘patriarchal femininity’ and ‘femmephobia’ operate by attempting to ‘turn an active (femme) subject into a passive object’ (p. 100). Further, femmephobia can become internalised and result in the setting of self-imposed limits in a process of ‘naturalised feminine devaluation’ (pp. 101–102) [7]. Gendered violation and discrimination of femininities, ‘victim-blaming’, ‘slut-shaming’, as well as a narrow tightrope-walk between femininity constructs of ‘Madonna’ versus ‘whore’ are then understood as part of a commonly internalised, naturalised femmephobia [7].

The Swedish welfare state and its policies and ideals are an important contextual and historical frame for this study. The formation of the Swedish welfare state, also referred to as the ‘caring state’ or the ‘people’s home’ [20,45], can be linked to changes in women’s situation, societal position and health condition. In parallel, the feminist movement was influential [45,46]. Historically and ideologically, a strong focus on education and active labour market policies has underpinned the Nordic social democratic welfare program [20]. With nineteenth-century roots, an activist struggle for social, religious, and political rights was central to the Swedish welfare project. The forerunners of this struggle were ideas about social transformation of ordinary people through ‘social solidarity’ and ‘social responsibility’. These ideas included a belief in formal and popular education as a means to inform people about their duties, improve their social and economic situations, and influence population health [47]. These influential social movements promoted the idea of a ‘sober’, ‘proper’ and ’respectable’ worker through a collective social process of ‘discipline’, ‘cultivation’ and ‘civilisation’ [48,49].

This Swedish ‘respectability-project’ formed the basis for the political welfare ideal of upward mobility through higher education. For girls this meant being ‘good’, ‘clever’ and ‘effective’ in places like school [50]. The Swedish sociologist Sohl [50] connects the welfare ideal of Swedish women’s upward mobility through education to Skeggs’ [27] theorising of how British working-class women construct ‘respectability’ and ‘femininity’ as they strive to prove themselves worthy in congruence with the ideals of middle-class femininity. Skeggs [27] places the formation of class and gender in a historical context. By the end of the nineteenth century, ‘femininity was seen as the property of middle-class women who could prove themselves to be respectable through their appearance and conduct’. This is commonly coded as ‘passive’, ‘fragile’ and ‘dependent’ (p. 99). Femininity became a ‘classed sign’ through which white middle-class women gained superiority and distinguished themselves from those ‘inherently healthy, hardy and robust’ working-class women who consequently lacked femininity and respectability. Working-class women were associated with labour, and conduct such as being ‘excessive’ or ‘out of control’. In terms of bodily function, they were associated with sexuality and reproduction, and coded as ‘vulgar’, ‘pathological’ or ‘lacking discipline’ (pp. 99–100). This can be compared to Harris’ [9] theorizing of the contemporary construction of ‘at-risk’ girls who do not follow an individualised trajectory of ‘success’, but instead are connected to ‘failure’ as a ‘personal choice’ rather than circumstantial disadvantage (for example, in the school-to-work transition). Working-class women’s strong investments in femininity and ‘routes into respectability’ by appearing and being feminine, can be understood as a desire or struggle to avoid being connected to this negative coding [27].

Societal gender constructs and gender order not only shape young women’s opportunities in life, but also their agency and health [5,8,10]. Research is important but still lacking on health experiences related to the multitude of femininities across the lifespan and in various and changing social contexts. This study addresses this gap.

## Methods

A qualitative and longitudinal approach was chosen, and interviews were drawn from the Northern Swedish Cohort (NoSCo) [51]. Repeated interviews across age and time were used to empirically study constructs of youthful working-class femininities in a sample of unemployed and marginalised women in Northern Sweden during the 1980s and 1990s. The study was started in the early 1980s and therefore the results may be viewed as historical. Those who were young women at the time of interviews are now in their 50s, and that period of their youth represents an important transitional period from adolescence into adulthood, including the expected school-to-work transition.

### Social context

The setting was a medium-sized, residential, industrial municipality in northern Sweden between 1981 and 1998. At the time of the study, the municipality had about 70,000 inhabitants and was representative of Sweden as a whole with regards to socio-demographics, health status, and health behaviours among young people [51]. During the 1980s, this area was characterised by higher workforce immigration from Finland than the rest of Sweden, and comprised national minority groups such as the Samis (the indigenous population in Sweden), Swedish Finns, and Tornedalers (Meänkielä-speaking/Finnish-speaking people along the Torne River in northern Sweden) [52]. After the initiation of the cohort NoSCo, refugees from the Middle East, Africa, Asia, Latin America, Eastern Europe, and the Balkans have moved to northern Sweden.

Except for an endemic high unemployment rate that was twice as high as the country as a whole during the 1980s, the town was also representative of labour market conditions [51]. The labour market was extremely gender-segregated and male-dominated in the steel industry, technical university college, and Swedish Armed Forces. The public sector was dominated by women. In addition, there were specific state-driven projects that targeted young women to take employment in male-dominated market sectors [20]. An aim of the Swedish active labour market policy during the early 1980s was that no one under the age of 21 should be unemployed. In spite of these efforts, some young people were unemployed because there was a shortage of labour market programmes.

At the time, all schools were public, had no fees or other costs, and provided hot lunches. All classes were mixed in relation to gender. All pupils followed the same class through nine years of compulsory school. School reform, introduced in 1962 as part of the Swedish welfare state, afforded all children the right to nine years of basic education regardless of geographical location. Only a minority of Swedish teenagers completed secondary education in the 1960s. Completion increased in the 1970s and thereafter [53]. After ‘mother tongue reform’ in the 1970s, children from national minority groups were permitted to speak their native language in school (e.g. Sami, Finnish, Meänkielä) [52].

The municipality is located in Norrbotten, the northernmost county in Norrland. Norrland represents almost two thirds of Sweden’s land mass but the inhabitants constitute only 12% of the Swedish population [54]. Political rhetoric has long portrayed this remote and sparsely populated northern region as ‘problematic’, ‘in crisis’, and in ‘decline’ [54]. For several decades, young people, particularly young women, have moved from the ‘rural’ and ‘remote’ north to major towns or the capital city in the south [55]. In addition to economic reasons such as higher education, employment and income, a ‘macho culture’ is suggested as one possible factor for the out-migration of young women from the north [55].

### Design and participants

NoSCo is a prospective longitudinal cohort study [51] that consists of all pupils (n = 1083) who were in their last year of compulsory school in 1981 and attending any of the nine municipality schools. Cohort participants were interviewed at the study start and at each follow-up. A social medical approach was used, meaning that health and social context were explored from the vantage point of the young people. Information was gathered from participants who did not continue into secondary high school or had no job in early autumn 1981. All girls who were not in school, employment or training (NEET: youth not in employment, education or training) [56], were selected for this qualitative study. None was excluded. All but one individual participated in each of the requested interviews during the follow-up period.

Repeated interviews were conducted with each of these seven girls/women over 15 years, from 1982 (aged 16–17 years) until 1998 (aged 32–33 years) [57]. One woman was interviewed five times, while the others were each interviewed three times. In total, 23 personal interviews were conducted. All participants came from a working-class background, no parents had higher education, and parental unemployment was high. All of them had low school grades and no secondary or higher education. One was born in Finland while the others were Swedish born. According to the ethical standards of that time, no background questions were asked about sexuality, gender identity, native language, or belonging to an ethnic minority group.

### Data collection

Individual semi-structured interviews were conducted in Swedish by the NoSCo principal investigator (last author, AH), who is a family physician familiar with the setting, specialises in social medicine and gender perspectives in public health, and has been responsible for follow up interviews since the project began. The interviews lasted 30 to 60 minutes, and were supported by an interview guide with open-ended questions that probed the participant’s life situation, work or unemployment, leisure time, financial situation, family and other social relations, health and wellbeing, prospects that influence one’s life, and plans for the future. Health was asked about in open-ended questions, and measured with questionnaires reported elsewhere [51]. Follow-up at age 32/33 years focused on health-promoting experiences during their lives. Questions about gendered experiences, such as thoughts about being/becoming a mother, division of domestic work, demands and hierarchies in working-life, or abuse in partner relations were also a focus. The follow-up interviews were of a retrospective character, and included reflections on how life had turned out. These were compared to interviews during teenage years, when participants were asked to reflect about the present and future. Participants gave their informed consent before the interviews. Notes were taken after the interviews to capture the context. All interviews were tape-recorded and transcribed verbatim before analysis.

### Data analyses

The interviews were subjected to qualitative content analysis [58] that began with an inductive phase and was followed by a more theoretically informed phase. First, each interview was read and summarised to obtain a naive understanding of the manifest and latent content. This was followed by detailed inductive coding, in which the text was divided into meaning units that were condensed and labelled with one or several codes or short sentences. Open Code software [59] was used for the detailed coding and the clustering of codes with similar content and meaning across interviews. Next, we defined various femininities (categories) based on these clusters (subcategories). Guided by our theoretical frame, we defined ways of ‘doing’ femininity in relation to participants’ actual handling of situations, how they and people in their immediate surrounding acted, and their thoughts and attitudes. For example, *normative femininity* was understood to be characterised by ‘caring’ and ’respectable’ (seen by others as ‘good’ and ‘effective’), whereas *norm-breaking femininity* challenged this ideal. We moved between parts (codes/sentences/clusters) and our sense of the whole (interviews) to define the various femininities. The various femininities spanned the interview material, and thus codes and content from the same participant (at different ages) could be included in more than one constructed femininity. Related to participant social context and situation, we interpreted some aspects of the femininities as reflecting enhanced power, agency and health, whereas other aspects reflected diminished power, agency, and health (see the theoretical framework of ‘agency within structures’). Thus, we interpreted context-related content representing social aspects and structuring conditions as enhancing or diminishing agency and health. Social aspects included presence or lack of social support and gendered norms.

Our analysis yielded one overarching theme, and three femininities (categories) with interrelated subcategories. Quotes are drawn from each of the participants in the results, but in order to maintain confidentiality no pseudonyms or numbers are used.

## Results and reflections

The participants shared a working-class context, including less positive experiences in school, a drug-culture, the presence of violence, being dependent on parents or employment agencies, and having difficulty time getting a job or education. They also shared the wider socio-political context [21]. Within this context, the participants constructed *normative and altruistic, norm-breaking*, and *troubled* femininities during late adolescence and into young adulthood.

### Constructing respectability from disfavoured social positions

The overarching theme of ‘constructing respectability from disfavoured social positions’ reflects the struggle for self-respect and the respect of others, and their construct of different and dynamic femininities to achieve this respectability despite their lack of formal education and social status. Congruent with Skeggs [27], their ‘respectable self’ was constructed through many practices, processes, and techniques (p. 72). These various femininities are not static positions, but context-bound and dynamic, ongoing constructs and processes that are dependent on time, place, and structuring conditions. The social context, including social support and recognition, is seen as essential to whether the process promotes or lessens health. For example, solid support from parents during early school years was interpreted as enhancing overall health, respectability, and power to act, even during hardships and potential health risks such as early pregnancy. Poor support or violent relationships were construed as reducing power and undermining health and respectability. Social support was clearly an important buffer for life stressors [60,61].

### Normative and altruistic femininity

Participants who constructed *normative and altruistic femininity* claimed to have had a good life and to be in fairly good health. The main ailments mentioned were back and shoulder pain. These were linked to heavy lifting on the job or pregnancy-related issues. The strong influence of hegemonic gendered norms (caring and collective responsibilities were coded as feminine) was apparent in how participants constructed femininity and respectability as teenagers and young adult women. *Normative and altruistic* femininity was characterised by statements about taking responsibility for oneself and family, and within a larger social context was permeated by expressions that were coded as ‘relational and caring’, ‘labour-oriented’ and *altruistic*. This *normative femininity* construct is similar to the culturally dominant ‘traditional’ and ‘proper’ femininity described by Connell as an ‘emphasised femininity’, and by Blair and Hoskin [15] as a ‘culturally sanctioned’, ‘essentialised’, and ‘patriarchal femininity’ that takes heterosexuality and cisgender for granted and to be expected.

‘Relational and caring’ constructs of femininity were apparent in the first wave of interviews, when the teenage girls dreamt about romantic love and having a future nuclear family. Caring for others was a recurrent topic, and thus helping their mothers, friends, or animals was construed as a ‘natural’ way to act. This is consistent with Skeggs [27], who argues that becoming a ‘caring self’ is a ‘dialogic production’, meaning that ‘caring cannot be produced without caring for others’ (p. 56, 118–138). In this process, the girls saw their mothers as role models for behaving as respectable women and having a strong orientation towards the nuclear family. An unspoken assumption that the family would be heterosexual was conveyed by opinions like ‘a family should consist of a father and a mother’. They performed and prepared themselves for life in accordance with ‘becoming respectably heterosexual’ [27]. In this way, they were socialised and policed into ‘compulsory heterosexuality’ [38] and ‘the heteronormative matrix’ [39] early in life.

The participants’ labour-orientation reflected labour market initiatives focused on women’s paid work [20]. Having a job was understood to be a path to maturity and acceptance as a respectable and responsible adult in the community:

I mean, you can’t behave at work the way you might have at school – you can’t go around saying ‘Shut up’, ‘Fuck off’, and stuff like that. You just can’t, because you’ve got adult co-workers, so you have to be able to speak normally around them. I think working helps you mature – makes you think about things. (Woman 30 years)

Although they emphasised the importance of having a job, they maintained that after becoming a mother, the job should not be at the expense of their families. Family rather than the job was the basis for responsibility and identity, and expressed in statements like ‘My son means everything to me’. Caring for children was prioritised over a career or full-time work. This is in agreement with the discursive position that children’s psychological well-being is dependent on the accessibility of the mother [62].

In the labour market, they mostly entered women-dominated sectors as part of the gender-segregated labour market [63,64]. By balancing the norms of being a caring mother in a domestic field, and a good citizen who participates in the labour market, they could continue to behave as both respectable and caring [27]. A similar connection between a respectable feminine status and caring occupations was shown in a study by Huppatz [65]; working-class women in nursing and social work made great efforts to show the ‘right’ type of femininity that conferred social respectability. In that context, respectability status was perceived to increase with poor pay since it was connected to moral virtues such as selflessness. This position can be contrasted with that of the participants in our study who criticised the low wages in healthcare work as highly unfair, thus reflecting claims regarding workers’ rights [47]. At the same time, being *altruistic* was highly valued.

During the same time period, labour market policies prompted women to enter male-dominated work domains that were in need of workforce [20]. A clash between their own incorporation of ‘traditional’ gendered norms (hegemonic patriarchal femininity) and external societal demands and regulation of women’s ‘working-class bodies’ was revealed in this situation. One of the girls and her mother strongly opposed the recommendation that the girl to apply for work at the local steel company:

They [the employment agency] want girls to go after typical men’s jobs. They’d really like to see you applying to the Ironworks or something like that – I don’t think I would fit in. I think that would be more suitable for a guy. (Girl 16 years)

Lucas and Steimel [66], in a study of mining from the same period, described how gendered norms in the local community resisted women’s involvement in masculine blue-collar work. In response, women mine workers either distanced themselves from femininity in order to be respected as (masculine-coded) mine workers or exaggerated their femininity to be seen as ‘real’ women. Among our participants, mining was not part of constructing ‘respectable’ femininity. In comparison, but related to the context of late nineteenth century, Weitz [26] and Skeggs [27] point to the ‘paradox’ of paralleled, socially accepted ideas and classed/gendered constructs of women’s bodies: an emphasis on the ‘frailty’ of middle-class women’s bodies versus the ‘robustness’ of working-class women’s bodies (both white and non-white).

*Altruistic femininity* was constructed through participant engagement and care for others who were unable to take care of themselves, such as the mentally ill, disabled, aged, workmates who were afraid to speak up for better working conditions, or their own families. Being *altruistic* meant fighting for people around you without financial gain. In such situations, the participants demanded space and expressed strength and determination without fear:

I’m active in the union and I think we need to stick up for our rights./…/Healthcare jobs are too low-paid. I feel sorry for people who are too timid to speak up and say what they’re good at. They lose out salary-wise all the time. (Woman 30 years)

This *normative and altruistic femininity* construct corresponds to Skeggs’ [27] theorizing on ‘respectable femininity’, as well as to notions of the ideal ‘respectable worker’ [49]. As exemplified above, the influence of working class and social movements’ ideals were also strong, and expressed in statements about proving your worth through work and taking responsibility, and in the stress of being well and fairly treated [27,49,50]. This femininity of a ‘caring self’ can be understood as performed within the ideals and structures of the ‘caring state’ and closely tied to the Nordic welfare model [20,47]. At the same time, the construct was aligned with ‘essentialised’ and ‘naturalised’ femininity [7] and located within the heterosexual matrix [39]. Resistance against being used as ‘working-class bodies’ in (male-dominated) mining sectors was displayed because this risked undermining their respectability and femininity. In contemporary individualised discourses of personal freedom and ‘success’, young women’s orientation to altruism and care of others is seen as strong. At the same time, they must navigate traditionally masculine domains of work and study that are also classed and raced [10,67].

### Norm-breaking femininity

*Norm-breaking femininity* was represented by ‘rebellion and boundary-crossing’ as teenagers and by either being ‘independent’ or ‘forced to be strong’ as young adults. As teenagers, positions associated with ‘rebellion and boundary crossing’ femininity were either being ‘rebellious, strong and respected’ or ‘rebellious, risk-taking and destructive’. These positions differed in terms of respectability and health experiences.

‘Rebellious and strong’ is interpreted as enhancing respectability, power and agency. This position is characterised by culturally sanctioned, ‘healthy’ boundary-crossing and resistance, and reflects the emergence of influential activist and feminist movements towards gender equality and women’s independence. This was in parallel with workers’ rights enacted by the dominant and masculine-dominated workers’ unions at the time (also seen in the construct of *altruistic femininity*). The issues raised related to influencing decisions in school or work, or feelings of being good enough among peers without having to behave as others do, and expressed as ‘if I’m not good enough as I am, so be it’. This demonstrates awareness of inequities as well as their personal value and rights, and how the culturally sanctioned social movements of workers’ and women’s rights facilitated transgression of normative femininity constructs. Those who constructed respectable femininity through a labour-orientation strongly opposed being ‘used’. For example, they refused internships that would have paid them lower salaries than other staff performing the same work tasks, saying: ‘I don’t think it’s right not to pay interns. It’s like they’re making the intern into their slave’. On the other hand, as described earlier, the ‘tightrope walk’ between conforming to or transcending hegemonic femininity constructs was difficult if respectability was to be preserved in the eyes of oneself and others.

As teenagers, some participants described themselves as ‘coltish’, ‘goofy’, ‘prankish’, ‘rowdy’ or ‘aggressive’. They broke away from the hegemonic and patriarchal norm for well-behaved ‘nice’ and ‘good’ girls. For example, they might have had frequent school absences and truancy. Respectability was mainly constructed in relation to peers. Some acts of rebellion were interpreted as ‘destructive’ because they involved risk-taking, violence, heavy alcohol or drug intake, or being careless in sexual relationships, and undermining their social situation, respectability and health. Thus, these were not culturally sanctioned. When 15 years old, one left her foster family after many conflicts, and lived outside of her parents’ control. This can be understood as a way to demonstrate agential capacity and resilience to survive a destructive family situation. These girls said that they were seen as ‘black sheep’, ‘gangsters’, or just deviant and different. Their acts and constructs exemplified constructs of ‘failed femininities’ that oppose the (white) patriarchal hegemony [7]. Because their gendered performances were signs of ‘culturally unsanctioned’ behaviours, they were exposed to policing and devaluation, in accordance with descriptions of ‘femmephobia’. According to Hoskin [7], femmephobia works to turn active (femme) subjects into passive objects within a hegemonic gender system of ‘proper womanhood’. The negative naming serves as an example of ‘ascribed’ femmephobia [7], a ‘linguistically embedded’ way to devaluate and subordinate ‘deviant’ and transgressing femininities in daily life.

During teenage years, norm-breaking had consequences for completion of schooling. The consequences of sexual risk-taking and unwanted pregnancies varied depending on the support available in their immediate social context. Parents had an important role, whether supportive, patronizing, ‘blaming and shaming’, or merely absent. Their behaviours may be interpreted as performance of the ‘excessive’, ‘vulgar’, and ‘out-of-control’ working-class girl script [27], and is reminiscent of the struggle within the workers’ movement between the more ‘wilful’ workers who refused to adapt to the ‘respectability project’ and the more ‘proper’ and compliant workers [48,49].

‘Independent femininity’ among young adults is associated with the constructs ‘refuse subordination’, ‘setting limits’, and ‘forced to be strong’. Respectability (by independence) was constructed in a more individualistic way than *normative and altruistic femininity*, and mostly by the women who were rebellious when young and continued to be rebellious as adults. Those who refused subordination constructed their independence in relation to parents, the youth employment office, and men as partners. Those who constructed independence went their own way, and this meant that they had longer periods of unemployment and became single mothers and sole breadwinners for the family.

I think it’s better [that] I live like a lonely mother. It’s better than having a dad coming and bothering when they want the kids. Oh…I think it’s better to be alone. Because we have our rules. There is no one else who is opposing (them), if I say so, yes… one hears so many relationships are breaking and there should be shared custody…and Carl [son] sometimes asks who his dad is and I tell him who it is and then it’s fine. (Woman 32 years)

As a downside, the woman above became lonely and isolated in her parenting, and the loneliness sometimes resulted in nightlife that involved drinking and smoking. This contrasted to the hegemonic ideal of a ‘worthy’, healthy and responsible female citizen [68] and the ideal of a sober, proper and respectable working-class woman [49]. These actions are understood to challenge traditional expectations of women’s heterosexual partner relationships and working life, but also had negative health consequences because of poor social support.

As a consequence of hardship in life, or consistent with their opposition to patriarchal hegemonies of ‘soft’ and ‘nice’ femininity constructs, some participants constructed themselves as ‘tough’, and capable of setting limits and defending themselves. However, their narratives also revealed low self-confidence and vulnerability:

I have this firm sense of my own boundaries and I’ve had it ever since I was bullied in school. It’s like ‘This far and no further’, or ‘Don’t come any closer or I’ll whack you’ … But if someone wants to hurt my feelings, they can. (Woman 30 years)

When linked to earlier experiences of being bullied and violated, their insistence on limits and strength can be understood as a way of upholding respectability, agency, and control in their own eyes as well as in those of others, despite life situations that threatened to destroy their self-confidence. This femininity construct demonstrated resilience, integrity and agential capacity.

Another expression of coping with experienced hardship was illustrated by one woman who struggled with dyslexia and was bullied at school. Afraid that her daughter would face the same obstacles, she demanded extra help for her daughter in school. At the same time, she reproduced the ‘forced hardness’ in her treatment of her daughter:

She (my daughter) hardly dares to come home if she’s done something stupid at school, because she knows I’ll be on her case immediately. (Woman 30 years)

Because of subordinated social positions and lack of support, they were either *forced to be strong* as part of constructing respectability, despite being devalued and violated, or they showed strength as part of their ‘empowered femininity’ in opposing or challenging masculine hegemony [7]. This can be referred to as the discourse of ‘the strong Nordic woman’, who despite exposure to oppression and men’s violence, is expected to show strength and cope on her own without culturally sanctioned social support [69,70]. This forced form of marginalised and independent (and occasionally violated) femininity was associated with several aspects of ill-health, although leaving an oppressive man was perceived as a release and health-promoting. Living alone and upholding a strong façade of hardness as a defence against critique and femmephobia was occasionally accompanied by self-destructive behaviours like smoking, drinking, and avoiding medical consultations.

### Troubled femininity

*Troubled femininity* was constructed by insecurity, or even suppression and violence, and by wistfulness when (as young adults) participants looked back on dreams that never came true. In contrast to the *normative and altruistic femininity*, the *troubled femininities* were associated with low social support, feelings of doubt, low self-worth, experiences of illness, and depression. However, *troubled femininity* was also a construct of resilience, survival of hardship and marginalisation, and included hope for the future.

During the teenage years, construction of an ‘insecure femininity’ comprises identity-trouble, mixed-up feelings, feelings of being an ‘outsider’ and doubting one’s future. Identity-trouble was understood as being at a ‘breaking point’ in life, ‘hard to find out who I was and what I wanted’, or ‘being both grown-up and a child’ in others’ eyes.

When unemployed as a teenager, distress and a wide range of health problems were expressed, as well as pessimism about the future. One of the girls worried about ending up as a ‘homeless alcoholic’. This can be interpreted as a fear of losing respectability:

Sometimes I can’t sleep, and I used to have a lot of headaches … you sit and worry and get a headache. Sometimes I get a stomach ache, stomach problems can be from nerves… I’m restless, I’m nervous about the future and I think: ‘Should I sit here like this? … Am I ever going to get any job? … Am I going to have to wait for years until I get a job?’ Sometimes I don’t think about it at all. I just think about having fun with friends and stuff … but when I do think about it I get depressed and think ‘Am I going to turn into some kind of homeless alcoholic, end up as a drug addict?’ You don’t know what you could become … What is going to become of me? (Girl 16 years)

Insecurity when young was also referred to as general feelings of being ‘uncomfortable’ or ‘an outsider’ in social situations, and not being able to live up to demands and expectations of ‘successful’ femininity. When asked why she did not visit the employment agency, one of the girls answered:

I don’t know, I have enough with my dog… so they should contact me. I am so extremely shy and have trouble being in contact with people. When I sit by strangers, then I can be quiet all night, and not say a word. It feels stupid. (Girl 16 years)

When asked what they thought they would be doing in ten years’ time, several doubted they would have a job or a profession. They therefore down-sized their plans and compromised their dreams. This can be understood as self-regulation within limiting gendered and classed structures. As young adults and mothers, their pessimism was expressed as a sense of age discrimination, as ‘employers would rather hire adults with grown-up children’. The constructs of ‘insecure femininity’ have similarities with Skeggs’ description of an ‘ambivalent’ femininity [27] (pp. 98–99).

The ‘suppressed and violated femininity’ was built on disfavoured positions in heterosexual relationships, manifested in experiences of various kinds of abuse, and demonstrates exposure to masculine dominance and regulation, including femmephobia [7]. Despite her young age (13–14 years old), one girl’s first boyfriend beat her and forced her to have sex. Thanks to a good relationship with her parents, she told them about the abuse and they immediately reacted and stopped the destructive relationship. When, at the age of 33, she was asked whether the abuse could explain her poor health status with pelvic infections, she said:

Yeah, it can be like that, I don’t know, because it’s always there, I mean, stuff you never forget, it’s always there. (Woman 33 years)

After a violent relationship, another woman suffered both physical and emotional injuries resulting in depression and suicidal thoughts:

I’ve had a really rough time. Sores and things heal, but emotional scars – they last. They are harder to mend. I will never be able to completely trust anyone again. (Woman 30 years)

Exposure to violence contrasts with dreams and plans of romantic love and stable relationships, although both aspects (theoretically) can be understood as integral in a hegemonic gender system of regulatory heteronormativity [39] and patriarchy [7].

Construction of a ‘wistful femininity’ reflects participants’ feelings as young adults, looking back on their lives, while also planning and hoping for the future after resisting and surviving hardship in life. They expressed regret, sorrow and sadness at choices they had made as teenagers and wished they had done otherwise. Participants who had a good life as adults, and those with more problematic life situations, expressed similar regrets about leaving school early, indicating that ‘one has to get educated, without going to school, one isn’t worth anything’. Over the years they had learned that good jobs demand proper education, and they tried to convey this to the next generation:

Finish school – that’s something I will insist on for my own kids – to make them want to keep up with their school work. ‘Cause that’s something I regret … I threw away so many years of my life for nothing, really. (Woman 30 years)

Constructs of wistfulness were also tied to ‘dreams that never materialised’. When as young adults they reflected on their dreams, the financial burden of several years of study was perceived as an insurmountable obstacle. Dreams of becoming a psychologist, music teacher or occupational therapist had been set aside because they were thought to demand too much effort from those who had to start with getting better grades in ordinary school. In this way, they kept to the narrow tightrope of femininity that intersected with being born into a working-class family with limited resources.

Broken dreams about the ‘lucky’ nuclear family, in line with normative constructions of ‘successful life-trajectories’ [9] and ‘respectable heterosexuality’ [27], were also expressed by women with broken relationships. One woman, who was given up for adoption as a baby and left her family at age 16 after a conflict with her stepfather, dreamt about starting over again. This way, she put herself in the centre, transgressed, and planned for the future by turning her back to the negative consequences and expectations of normative and patriarchal femininity:

I feel I don’t belong to any family. I’d like to change my surname and start a whole new family tree starting with me and my girls, so I would feel we are the way we are now. (Woman 30 years)

Health problems related to wistful femininity were stress and depression due to emotional strain, grief over broken relationships and loss of life chances. There were also physical ailments in the form of sleeping problems, shoulder pain, and low back pain. However, these participants emphasised coping and adaption to their life circumstances, regardless of whether or not they were content.

On the whole, participants who constructed a *troubled femininity* lacked recognition. In this sense the troubled position differed from the construct of *normative and altruistic femininity*, which was constructed in relation to a ‘respectable’ occupation and reciprocal social relationships with their own mothers as role models, and support for their (caring) feminine position in the wider society. Recognition is seen as a significant moment in the construct of subjectivity. According to Skeggs [27], the ability to engage in this dialectic is a matter of social position. On the other hand, these women demonstrated several signs of ‘empowered’ and ‘transgressive’ femininity constructs, as well as agential capacity and resilience within the structuring conditions of marginalisation and hegemonic patriarchy in society at the time of their transition into adulthood.

## Discussion

This study explores the construction of multiple and dynamic femininities in relation to structuring living conditions and health experiences among a group of marginalised 16-year-old girls, unemployed and without further education, as they develop into young adult women.

Our key findings elucidate how participants strove to construct ‘respectability’ from disfavoured social positions, and how these diverse constructs were tied to their experiences of health and social support. Although a bit simplistic, the more they kept to the *normative and altruistic femininity*, the more social support and health they gained, compared to if they challenged this ‘essentialised’ or ‘patriarchal’ way of doing femininity or failed to conform. In general, social stressors and discrimination stressors have a damaging impact on physical and mental health, and social support is proven to be an important buffer for life stressors [60,61]. Thoits [60] concludes that differences in exposure to discrimination and stressful experiences is a ‘primary way that gender, racial-ethnic, marital status, and social-class inequalities in physical and mental health are produced’ (p. 41), and that such exposures widen the health gaps between advantaged and disadvantaged groups. Within a colonial framework, Hoskin [40] points to the gendered devaluation at play when contrasting the ‘failed femininities’ of native and indigenous women who fail to comply with the coloniser to the ‘superior femininities’ of European women. ‘Failed femininity’ is described by Hoskin [7] as the ‘failure or refusal to approximate the patriarchal feminine norm of white, cisgender, able-bodied virtuosity’ (p. 100). Such notions about social support versus social devaluation among our participants may help to understand differences in their experiences of health and illness.

Health-promoting processes in the construction of a *normative and altruistic femininity* were tied to a caring and relational orientation, taking responsibility, and being labour-oriented. The construct was tied to good health and few health problems, suggesting that the women experienced a balanced life situation as well as good health in congruence with societal and patriarchal expectations. Research suggests that caring and helping others often has a greater impact on women’s mental health than on men’s [71]. The ability to help and care for others, both characteristics of ‘emphasised femininity’ [37], may counterbalance a number of negative factors in a job; inability to care for others may have devastating effects [71]. On the other hand, in gender unequal relations, caring also has negative consequences in terms of heightened workload and job strain in multiple arenas [10,64]. In the context of youth mental health and stress, Wiklund et al [10] problematise the regulative normative and demanding expectations on contemporary young women’s caring and responsibility-taking (both in domestic and public fields) in parallel with low social support and recognition for achievements in school and working-life. Our results clearly show that femininities that transgressed normative expectations in various ways were less valued, and impacted health and opportunities. In some respects, although found in diverse social contexts, the normative femininity in our study has similarities with the construct of ‘conservative femininity’ (contrasted to a more ‘modern femininity’) defined among South African teenage women [8]. The construct of conservative femininity implied acceptance of unequal gender relations and domestic arrangements, and expectations of passivity in relation to masculine domination. In their study, as well as in ours, there were also ‘femininities in transition’ who challenged masculine domination, control and violence and strove for respect [8].

Hence, *norm-breaking femininities* could be ‘respectable’ and health promoting by constructions of being rebellious and strong, and demonstrating resistance to traditional and ‘conservative’ constructs of girls and women as entirely ‘nice’ and ‘there for others’ [34]. Coming from subordinated social positions, and despite lack of social support, the women constructing *norm-breaking* and independent femininity were forced to show strength to uphold their respectability as worthy mothers and equal women. This is congruent with the discourse of gender equality that permeated mid-1990s Swedish society. However, *norm-breaking* and risk-taking femininities could also be destructive for the girls’/women’s health status. These young at-risk women represent those who ‘fail’ to transfer from a disadvantaged social class and can be compared to the negative coding of working-class women as ‘pathological’, ‘vulgar’ or ‘out-of-control’ [27], and to Harris’ [9] conceptualisation of girls ‘at risk’. Girls’ ‘antisocial trajectories’ are less often addressed than boys’ are, but as indicated in our study, can involve sexual victimization, troubled partner relationships, and difficulties parenting their children [72]. Navigating constructs of ‘proper’ traditional and patriarchal femininity versus women’s liberation may have influenced the young women, particularly as teenagers. As Hoskin [7] states, ‘patriarchal femininity’ requires that one ‘walk a very narrow tightrope’ of the ‘Madonna-whore’ construct and its risk of facing ‘slut-shaming’ (p. 103). Thus, the dominant societal discourse of caring and constructing ‘nice’ femininities within heteronormative relationships and nuclear families that was present at the time for the study and including presumed ‘femmephobia’ [15], did not facilitate the opening of space for alternative and ‘empowered’ femininities that could gain public respect and wider social support. Instead, femmephobia worked to devaluate and police ‘culturally unsanctioned’ femininities that did not ‘uphold a patriarchal model of womanhood’ [7] (p. 101). Despite such regulatory discourses, our results highlight diversity regarding agential capacity and conformity to, or transgression of, culturally sanctioned femininities.

The social context behind the ill-health generating *troubled femininity* was related to perceptions of low self-worth, feelings of lost life opportunities, and severe mental illness. Another aspect of constrained agency and health was exposure to violence in oppressive heterosexual relationships as teenagers and young adults. This mirrors gendered patterns in the wider population of disfavoured labour market positions [1]. Strong links are found between violence, mental illness, and other health problems such as long-term pain and posttraumatic stress [73]. ‘Wistful femininity’ opened up space for reflection, resilience, empowerment and hope in thinking about possibilities beyond living in violent and masculine dominant partnerships, and ‘starting anew’.

One way to understand *troubled femininity* and the other constructs could be by turning to Walkerdine’s [74] problematisation of societal expectations of women’s ‘upward-class mobility’ embedded with ambiguity, anxiety and pain. In our study, these feelings were interpreted as wistfulness and sorrow about not being able to fulfil dreams. This may mirror participants’ ‘failures’ to live up to the welfare idea that everyone has access to upward-class mobility and social transformation through higher education [47,50]. Participants belonged to a group of women who did not move south for work or education [55,75]. These ideas about mobility and transformation also correspond to the ‘grand narrative’ about successful pathways in life being available to everyone despite social position [9,50]. In the context of upward-class mobility, Sohl [50] problematised women who either feel ‘at home’ or ‘out of place’ (p. 461). Applied to our study, the women either constructed normativity and ‘felt at home’ within the ideals and structures of the ‘caring state’ (despite a lack of higher education), or constructed *norm-breaking* or *troubled femininity* and felt ‘out of place’ in relation to the grand narrative of social transformation through education, respectable work and upward-class mobility. In this sense, participants walked a narrow tightrope in terms of constructing a ‘culturally sanctioned’ femininity and life-trajectory that could garner respect within the structuring conditions of limited material recourses and scarce social support.

Our results point to variations within the group of marginalised young women. Several potential axes of inequities and oppression [18] were understood to be at play, such as young age, gender, rurality, unemployment, working-class background, low social support, and undermined health (disability). These were in parallel to the narrow tightrope of constructing ‘respectable’ and culturally sanctioned femininity. Expression of ‘femmephobia’ [7] is understood as one additional ‘axis of oppression’ present in the young women’s narrations that was at work regulating their agency, femininity constructs, and life opportunities. This highlights the importance of addressing gendered life circumstances across the life course, young women’s struggles to cope and preserve respectability and agency despite disfavoured social positions and, in some cases, ‘antisocial’ trajectories or other life choices in synchrony with normative and patriarchal femininity constructs. This study is a call for more research on health experiences related to the multitude of constructs of femininities in various social contexts. In the early 1990s, there were many calls for more knowledge about women’s health and illness [1,76]. Despite the successful development of gender research in public health, we still lack knowledge and research about how femininities are constructed in relation to health experiences in various groups of women and various contexts.

### Implications for policy and practice

In the development of gender-sensitive interventions, for example in the context of youth mental health [72,77], we suggest that girls’ and young women’s constructions of femininity and ‘respectability’, including their agential capacity and strategies to uphold self-worth, be acknowledged. Our results point to the importance of contextualising girls’ and young women’s health problems, including ‘femmephobia’ and the consequences of subtle or tangible systemic devaluation and violation of femininity [7,12]. Future studies and interventions need to embrace the whole multitude of femininities in terms of genders, sexualities, ethnicities/race, (dis)abilities, and social positions in society, and include ‘empowered femininities’ and ‘femininities in transition’ [8]. Gender-sensitive interventions need to strengthen girls’ and young women’s position in society, and prevent gendered exposure to violence.

Gender, class, power relations, education, position in the labour market, and working conditions are known to be important social determinants of (in)equity in health within local and global contexts [11,24,76]. In a global health context, basic and secondary education is an important social determinant of adolescent and adult health in both poor and rich countries [78]. The beneficial effects of education can be attributed to the better living conditions that become available with education, including better jobs, higher income, and more control over one’s life circumstances [79]. As exemplified in the present study, lack of further education is negatively related to life-trajectories and associated with poverty, gender inequality, drug-use, teenage pregnancies, intimate partner violence and various health problems [80]. However, inequities in work-related health and sickness-absence, including temporary employment and ethnic discrimination for immigrant women in Sweden and elsewhere, show why gendered knowledge is needed [81,82].

Policy-making and interventions need to address the fact that young people’s mental health is related to their position in the labour market, as they may be unemployed or temporary employees [1]. In the Western world, rates of unemployment and mental health problems are increasing [83,84]. One of the few reviews of the gendered health consequences of youth unemployment highlights some important cornerstones in the construct of gender in relation to health among long-term unemployed working-class young men and women [1]. These cornerstones are gender-based violence in society (including violence in close relationships), women’s subordination in society, and which gendered expressions of feelings (such as low self-worth and depression) and health behaviours are legitimate for men or women [1]. In the present study, depression and low self-worth were more often tied to *troubled* and ‘violated’ femininity, although participants also expressed agency and resilience within structuring conditions such as heteronormativity and marginalisation in working-life.

### Methodological and theoretical reflections

This study has strengths and limitations. To strengthen trustworthiness, the analysis was continuously compared and discussed by the research team in accordance with the process of triangulation [58]. Because this study consisted of a small sample of seven women, there is limited potential for generalizing the results to a larger group of women. However, the repeated interviews and our theoretical point of departure gave us the opportunity to theorise and contextualise our findings in light of prevailing living conditions. Despite the small sample, this contextualisation increases the opportunity of relating the findings to how other groups of socially disfavoured young women construct femininity. Nonetheless, it is crucial to remember that processes of ‘doing’ femininity are bound to context and therefore may change with historical and political context [28]. Our results reflect a specific group in and context of northern Sweden in the 1980s and 1990s.

The population and longitudinal design of this study are unique in its long-term follow-up and selection of a disfavoured group of early marginalised young women who would probably be non-responders in most cohort studies. Such a prolonged engagement is unusual in qualitative studies. A strength of this study is the methodological approach, with repeated interviews over nearly 20 years and at different ages, because this provides insights into aspects of ‘continuity and change’ in the young women’s social lives and gender constructs [57,85]. A challenge in future research is to continue the interviews while focusing on the period from the 2000s to present. Completion of longitudinal interviews up to the present may enhance our understanding of femininity constructs and health development over the life span, and during a period of rapid societal change and gendered patterns of mobility in a remote northern region that is historically viewed as in crisis and decline [54,55,75].

Methodological and theoretical limitations include the lack of research questions and demographic information about gender and sexualities beyond ‘cisgendered female bodies’ [41] and ‘normative white femininities’ [17]. There may be participants in the sample who represent sexual and/or ethnic national minorities which were not explored. Such lived experiences may have added dimensions to the intersections of marginalisation through more explicit aspects such as low educational level, unemployment and undermined health (disability). This weakness mirrors the time when the data were collected, and the general marginalisation of such ‘deviance’. It may also mirror our own normative and biased lens and view as the research team [40], despite being gender and feminist researchers with a mission to challenge power and to reveal inequities in health and living conditions. A promising future direction in health research on gender and femininities is to broaden the scope toward truly intersectional approaches and critical femininity studies [7,15,17,40,41], and to explore health, lived experiences and social constructs beyond patriarchal white femininity. Further investigations of eventual general devaluation and discrimination of femininity in various social contexts, including ‘femmephobia’ and masculine privilege [15], are also important future analytical and theoretical directions.

## Paper context

Marginalised and unemployed young women have a vulnerable position in society and poor health. Few studies have investigated constructs of femininity in relation to health and living conditions among young marginalised women. The study explores how marginalised women construct *normative and altruistic, norm-breaking*, and *troubled* femininity; and how they strive to construct ‘respectability’ from disfavoured social positions. Gender-sensitive interventions need to strengthen girls’ and young women’s positions in society, and prevent exposure to violence.
